# Osteogenic and Neurogenic Stem Cells in Their Own Place: Unraveling Differences and Similarities Between Niches

**DOI:** 10.3389/fncel.2015.00455

**Published:** 2015-11-24

**Authors:** Wanda Lattanzi, Roberta Parolisi, Marta Barba, Luca Bonfanti

**Affiliations:** ^1^Institute of Anatomy and Cell Biology, Università Cattolica del Sacro Cuore, Rome, Italy; ^2^Latium Musculoskeletal Tissue Bank, Rome, Italy; ^3^Neuroscience Institute Cavalieri Ottolenghi (NICO), Orbassano, Italy; ^4^Department of Veterinary Sciences, University of Turin, Turin, Italy

**Keywords:** brain repair, neurodegenerative diseases, neural stem cells, mesenchymal stem cells, adult neurogenesis, osteogenesis

## Abstract

Although therapeutic use of stem cells (SCs) is already available in some tissues (cornea, blood, and skin), in most organs we are far from reaching the translational goal of regenerative medicine. In the nervous system, due to intrinsic features which make it refractory to regeneration/repair, it is very hard to obtain functionally integrated regenerative outcomes, even starting from its own SCs (the neural stem cells; NSCs). Besides NSCs, mesenchymal stem cells (MSCs) have also been proposed for therapeutic purposes in neurological diseases. Yet, direct (regenerative) and indirect (bystander) effects are often confused, as are MSCs and bone marrow-derived (stromal, osteogenic) stem cells (BMSCs), whose plasticity is actually overestimated (i.e., trans-differentiation along non-mesodermal lineages, including neural fates). In order to better understand failure in the “regenerative” use of SCs for neurological disorders, it could be helpful to understand how NSCs and BMSCs have adapted to their respective organ niches. In this perspective, here the adult osteogenic and neurogenic niches are considered and compared within their *in vivo* environment.

## Introduction

Stem cells (SCs) are considered “functional states” rather than “cell types” with a specific morphology and function, these being features more typical of mature cells (Morrison and Spradling, 2008). SCs act dynamically in tissue development, renewal, and regeneration, their activity and fate being regulated by molecular and cell-to-cell contact signals from the surrounding environment. Hence, somatic SCs in adult organs live within – and need – highly regulated, morpho-functionally defined microenvironments known as niches (Scadden, 2014). During development and growth, these niches remain “trapped” within tissue architectures throughout the body. As a result, different niches populate the organs and display variations of a common theme, sharing features which “adapt” to different functional demands. In spite of a vast amount of research, it remains largely unknown how diverse SCs and their niches function *in vivo* within different organs. By contrast, *in vitro* research on SC biology has been characterized by repeated breakthroughs, resulting in the perception that SCs can easily cure many diseases (Bianco et al., 2013a,b; Cattaneo and Bonfanti, 2014). At present, however, only selected populations of adult SCs are able to repair a limited number of skin, cornea, and blood pathologies, being of limited use in other contexts. Despite a lack of reliable evidence, statements in the media and even scientific papers have emphasized the use of “mesenchymal” stem cells (MSCs) such as those residing in the bone marrow (BM) stroma, as a source of trans-differentiating elements capable of colonizing different organs (including the brain) to replace lost cells. On these bases, MSCs have often been presented as elements which could overcome the strict rules regulating the SC niche/tissue relationships, even if most of their regenerative outcomes have not been confirmed by subsequent studies, since “MSCs commonly defined by *in vitro* functions have entered clinical application despite little definition of their function in residence” (Park et al., 2012). In addition, MSCs are usually considered as the osteogenic SCs residing in the BM stroma. Nonetheless, the term “mesenchymal” is now considered inappropriate as these adult SCs are biologically distinct from the embryo “mesenchyme”; accordingly, they are called bone marrow stromal cells instead (BMSCs; Bianco and Robey, 2015). Beyond semantics, the sometimes confusing terminology used to define these cells reflects the complexity of their biology and the cellular heterogeneity of their niche.

The misunderstandings become even more astonishing if such cells are employed to heal neurological diseases, since the central nervous system (CNS), although hosting neural stem cells (NSCs), remains refractory to repair/regeneration (Bonfanti, 2011; Peretto and Bonfanti, 2014). This review outlines the state-of-the-art regarding the inherent specificity of osteogenic and neurogenic niches through a detailed comparison of the microenvironment housing stromal (osteogenic) and NSCs, as well as their outcome in physiological and regenerative conditions.

## Skeletal Stem Cells and Their Osteogenic Niches

Although bone biology is apparently understood, an unambiguous setting for the osteogenic niche still represents a conundrum, hardly unraveled even after extensive revision of the relevant scientific literature. Bones, as complex organs, in mammalian vertebrates involve distinct specialized tissues: bone, cartilage, adipose tissue, blood vessels, all derived from multipotent BMSCs, along with BM and nerves. Bone, as a tissue, is a specialized connective containing osteoblasts, osteocytes, and osteoclasts, which cohabit and maintain a mineralized supporting matrix. After birth, bones still grow to achieve the final size of the skeleton, through either endochondral (bone replaces a cartilaginous bud in long bones) or membranous (connective membranes in the skull vault are directly converted into bone tissue) ossification. Even beyond completion of ossification, all bones are still extremely plastic and capable of adaptation to mechanical forces and chemical stimuli: they increase their sizes through cortical modeling (bone apposition on external surfaces) and modify their shape through remodeling (coupled bone apposition and resorption). These processes persist in adulthood, though modeling activity significantly decreases after peak bone mass is achieved, with a chronology that varies in different species, due to the variable lifespan and mechanics (Hall, 2014).

Osteogenic niches are found throughout the skeleton. Although no data are available on their actual number, it is reasonable to consider each single bone housing an organ-specific niche: over 200 quite large niches orchestrate tissue remodeling to maintain stable biomechanical conditions upon changing environmental stimuli (Long, 2011), with mature lineages being homeostatically renewed on a monthly basis (Long, 2011; Park et al., 2012).

Given this complexity, a univocal definition of the proper osteogenic niche is still pending. Converging evidence indicates BMSCs as the most upstream progenitors in the BM stroma. They were initially described as an adherent, fibroblastoid cell population with inherent osteogenic properties (Friedenstein et al., 1970). Although cells sharing features with BMSCs are found in other tissues (e.g., adipose tissue and skeletal muscle; Asakura et al., 2001; Zuk et al., 2001; Barba et al., 2013), BMSCs represent the best characterized cytotype (Park et al., 2012), able to self-renew and to generate multiple mesodermal lineages found within a skeletal segment (Bianco et al., 2013a,b). A specific subpopulation of BMSCs – namely, skeletal stem cells (SSCs) – is thought to represent the direct osteogenic SCs giving rise to the osteoblast/chondroblast lineage (Park et al., 2012; Chan et al., 2013; Bianco and Robey, 2015; see below). Conversely, the osteoclast lineage derives from hematopoietic stem cells (HSCs) through differentiation of monocyte/macrophage precursors. The osteogenic and hematopoietic niches are functionally related and mutually inter-dependent within the BM environment in trabecular bone: BMSCs and SSCs support and regulate HSCs homing *in vivo*; HSCs provide osteoclast precursors that combine with osteogenic lineage’s cells to form bone structure (Morrison and Scadden, 2014).

Bone SCs are mostly found around the walls of BM sinusoidal vessels, close to pericytes, where they are thought to contribute to the formation of an “endosteal niche,” on the vascularized endosteal lining of bones (Sacchetti et al., 2007). SSCs also reside in the inner layer of periosteum, which is also highly vascularized and innervated (De Bari et al., 2006; Roberts et al., 2015); herein, they drive endochondral ossification, contribute to bone modeling and remodeling in both long and flat bones (Kronenberg, 2003; Chan et al., 2009), and are crucial for bone regeneration during fracture healing (Colnot, 2009). Therefore, two apparently separate compartments can contribute to the adult osteogenic niche: an inner “endosteal domain” – with BMSCs and SSCs housing BM cavities and lining endosteal surfaces – and a “periosteal domain,” being differently regulated and mediating different functions in bone homeostasis (Colnot, 2009). As periosteal vessels supply most of cortical bone vascularization, it is reasonable to consider blood vessels as the *trait d’union* between the two domains. Nonetheless, osteoprogenitors have been described also far from the typical perivascular location (Worthley et al., 2015).

The alternative ossification paths (endochondral and membranous), and corresponding embryo origins, suggest a regional segregation of niches (Schlecht et al., 2014). Most bones derive from the mesoderm through endochondral ossification, while skull bones originate from the neural crest (neuroectoderm), where highly migratory and plastic cells drive the membranous (direct) ossification of the skull vault (calvarium), coordinate skull–brain development and growth (Richtsmeier and Flaherty, 2013), and persist after birth within the dense connective tissue forming skull sutures (Lana-Elola et al., 2007; Lattanzi et al., 2012). Therefore, calvarial bone’s niches include endosteal and periosteal domains plus a “suture domain,” which progressively disappear as sutures ossify (Schlecht et al., 2014; Zhao et al., 2015). Moreover, the dura mater meninx underlying skull bones houses multipotent cells as external niche contributors (Opperman et al., 1993; Merrill et al., 2006).

Comprehensive descriptions of the skeletogenic lineage arising from BMSCs allowed identifying subtle immuno-phenotype and commitment-related differences within the lineage sequence (Park et al., 2012; Chan et al., 2013). Nonetheless, the criteria for univocal classification of SSCs as distinct from BMSCs are still unstable and pending. Both cells are perivascular, share stemness surface markers (see Table 1), and display extensive *in vitro* multilineage potential (angiogenic, adipogenic, and osteogenic), in spite of an extremely limited plasticity *in vivo* (Park et al., 2012; Bianco et al., 2013a,b; Chan et al., 2013). BMSCs typically display long-term self-renewal capacity, though they self-renew at a much slower rate compared to blood and epithelia (Kassem and Bianco, 2015). They commit to osteogenic precursors by expressing additional lineage-specific marker genes, hence turning into proper SSCs (Table 1). SSCs are mitotic, self-renewing, “oligopotent” elements, giving rise to cell progenies of bone tissue (osteoblasts and chondrocytes; Bianco et al., 2013a,b; Chan et al., 2013). Subsequent osteoblast progenies are endowed with an intense cell renewal potential and undergo relatively rapid turnover (Park et al., 2012). The entire and complex BM niche is maintained through constant interactions with vasculature and stromal components that regulate self-renewal and differentiation of SCs and early progenitors (Méndez-Ferrer et al., 2010; Ding et al., 2012). This structural dualism within the BM niche enables direct paracrine signaling between HSC and SSC niches: bone progenitors and osteoblasts provide regulatory cues for HSC homing and maintenance of hematopoiesis (Arai and Suda, 2007).

**Table 1:** **Common features and differences between osteogenic and neurogenic niches**.

	Osteogenic niche	Neurogenic niche
Types of niches	ALL BONES: *periosteal domain* (inner layer of the periosteum); *endosteal domain* (inner bone-lining and BM stroma) FLAT BONES OF THE SKULL: *suture domain* (within skull suture)	*V-SVZ* (lateral ventricle-olfactory bulb system) *SGZ* (hippocampus)
Number, location, distribution	*High number* (periosteal and endosteal niches are found in each skeletal bone); *Anatomically widespread* (in the whole bone)	*Very small number* (two main neurogenic sites in the brain) *Anatomically restricted* (ventricular lateral wall and dentate gyrus)
Types of stem cells (primary progenitors)	*Bone marrow stromal stem cells* (BMSCs; multipotent stromal, bone, cartilage, adipose, angiogenic progenitors) (αV integrin+, CD105+, STRO1+, CD45−; Tie2+; Nestin+) *Hematopoietic stem cells* (HSCs; multipotent blood cells and osteoclast progenitors) (CD45+, CD34+) (not considered here)	*Neural stem cells* (NSCs) *V-SVZ:* type B cells (radial glia-like cells, cilium) (Nestin+, GFAP+) astrocytic morphology *SGZ*: type 1 cells (radial glia-like cells) (Nestin+, GFAP+)
Number of stem cells	*High* (~12,000 clonogenicBMSCs through the skeleton of mice)	*Small* (~700 in the *V-SVZ*; the larger neurogenic site of mice)
Progeny and other niche contributors	PROGENY [Osteo-chondroblast lineage] *Skeletal stem cells* (SSCs; oligopotent – bone, cartilage, stromal progenitors; non-angio-, non-adipo-genic (CD105+, CD90+, Tie−); *Osteoblast progenitors* (CD90+, 6C3−, CD146+); *Osteoblasts* (metabolically active, OP+, OC+); *Osteocytes* (terminally differentiated, RANK L+ and ALP+) *Chondroblast/chondrocytes* (COL2+, ACAN+, SOX9+) [Osteoclast lineage] *Monocytes/macrophages* [from HSCs] (CD14+, CD33+) *Osteoclasts/osteoclast progenitors* (RANK+) LINEAGE SEQUENCE(S) Osteo/chondroblast lineage: *BMSCs* > *SSCs* > *osteoblast* or *chondroblast progenitors* > *osteoblasts* or *chondroblasts* > *osteocytes* or *chondrocytes* Osteoclast lineage: *HSCs* > *monocyte/macrophages* > *mononucleated osteoclast precursors* > *multinucleated mature osteoclasts* OTHER CONTRIBUTORS Stromal cells (6C3+; SDF1+), pericytes, e.c.m., endothelial cells, adipocytes, fibroblasts, nerve endings, dura mater (in skull bones)	PROGENY *V-SVZ*: *intermediate progenitor cells* (ASCLl+); migrating neuroblasts (PSA-NCAM+, DCX+) *SGZ*: *intermediate progenitor cells* (Type 2 cells) (Tbr2+, GFAP−, and mostly Nestin−) *Immature neurons* (*neuroblasts*) (PSA-NCAM+, DCX+) *Mature granule neurons* (functional nerve cells; NeuN+, Prox1+, DCX−) (also some *OPCs* and *mature oligodendrocytes*) LINEAGE SEQUENCE *Type 1 radial glia-like cells* (NSCs) > *intermediate progenitors* > *neuroblasts* > *immature neurons* > *mature neurons (some oligodendrocytes from OPCs)* OTHER CONTRIBUTORS Type 2 astrocytes (multipolar, GFAP+, S100β+, Nestin−), ependymal cells (in V-SVZ, facing the lateral ventricle), pericytes, endothelial cells, microglia, e.c.m. and fractones
Migration of the progeny	Osteoblasts, chondrocytes and osteoclastsdifferentiate locally, then migrate shortly during bone modeling/remodeling/healing Few osteoblast progenitors also migrate through blood circulation	*V-SVZ*: long distance migration in the olfactory bulb (mm in rodents; cm in primates); migration of OPCs into the white matter *SGZ*: short displacement within the dentate gyrus (up to hundreds μm)
Fate and final destination of the progeny	*Osteoblasts* at endosteum/periosteum-bone boundaries *Osteocytes* in interconnected lacunae (osteocyte syncytium) *Osteoclasts* in resorption (Howship’s) lacunae *Chondrocytes* on articular surfaces, in cartilage molds-epiphyseal plates during endochondral ossification, in cartilaginous callus at fracture site	*V-SVZ*: *olfactory interneurons* (at least six different subtypes) in the olfactory bulb; some oligodendrocytes *SGZ*: *granule cells* (glutamatergic neurons) in the granule cell layer of the dentate gyrus
Origin	Periosteal/endosteal niches derive from embryo *mesoderm*: both BMSCs and HSCs come from *MPCs* Skull niches derive from neural crest (*neuroectoderm*): SSCs derive from *neural crest stem cells*	Niches derive directly (V-SVZ) or indirectly (SGZ) from the periventricular, embryonic germinal layers (*neuroectoderm*) NSCs come from embryonic *radial glia* (transient type of astrocytes)
Regulatory molecules/pathways (in/on the niche)	Wnt/β-catenin, Ihh, FGF, IGF1, Twist1, RANK/RANKL/OPG, TGFβ, BMP-Smad, ERK, Ephrin, Kit-ligand, CXC-SDF, PTH/PTHrP, HIF1α, FoxC1, Heparanase, Kruppel-like factors 2 and 4, Hes4, Notch-Jag1 RunX2 common downstream transcription factor for most involved pathways Calcitonin, GH, PTH, PGE2, vit D3; sex hormones; cortisol; IGF; PDGF	*V-SVZ:* Wnt, BMPs, Noggin, IGF2, Shh (morphogens); EGF, FGF2, TGF-α, PDGF (growth factors); Notch, ASCLl/Mash1 (cell–cell interactions); GABA, Dopamine, Serotonin (neurotransmitters) Ephrines, ErbB4 and neuregulins *SGZ:* Wnts/sFRPs, IGF, BDNF, VEGF, EGF, IL4, IL6, IL1-β, TGF-β, TNF-α, GABA, Glu, dopamine, ACh, serotonin, leptin, estrogen, testosterone, corticosterone, endorphins
SC secretome [not considered here]^a^	NGF, BDNF, GDNF;VEGF, VEGFR, IGF1-2, NT-3, NAP2b, FGF, PDGF, HGF, SDF-1, SCF; CXCRs; proteins and miRNA (in microvescicles)	NGF, BDNF, GDNF;CNTF, NT-3, VEGF, FGFII, PDGF; proteins and miRNA (in microvescicles)
Relation/crosstalk with blood vessels	Perivascular localization of BMSCs, SSCs, osteoblast progenitors; IGF1, VEGF, PEDF, SDF1	Stem cells and transit-amplifying cells directly contact blood vessels; BDNF, IGF1, VEGF, PEDF, SDF1 (endothelial signals)
Rate of cell proliferation and progeny production	(Mouse endosteal niche) ~80% of endosteal BMSCs are clonogenic ~50% of endosteal osteoblasts are replaced over 14 days >80% of mature osteoblasts are replaced over 30 days	Less than 10% of Type 1 astrocytes (NSCs) proliferate ~87% of intermediate progenitors are actively cycling; they divide on average 3 times before differentiating; neuroblasts divide at least once ~10,000 new neurons are generated daily (mouse V-SVZ)
Homeostatic cell renewal	Rapid replacement of osteoblasts and osteoclasts *throughout the skeleton*, for bone modeling and remodeling (especially in periosteal domain); more active in long bones; limited in cartilage tissue	Neuronal replacement/addition *only within specific brain regions* (olfactory bulb and dentate gyrus); most of the CNS parenchyma is made up of non-renewing elements (apart from slow glial cell turnover)
Function of the finally differentiated cells	Matrix apposition (osteoblasts); mechano/chemo-sensing (osteocytes); bone resorption (osteoclasts); production of cartilage e.c.m. (chondroblasts/chondrocytes)	Learning, memory (V-SVZ, SGZ) Pattern separation (SGZ) (partially still unknown)
Modulation of activity by environment	Physical activity, mechanical loading, trauma (stimulatory) [for internal regulation (e.g., hormones, growth factors) see above]	Physical activity (stimulatory); running: >neuronal production environmental enrichment: >integration; stress, aging (inhibitory); [for internal regulation (e.g., hormones, growth factors) see above]
Changes in activity with age	BMSC division decreases in terminally formed vs. developing bones, then decreases in elderly (disappears in suture domain) *Endosteal niche*: active during bone elongation *Periosteal niche*: rapid expansion at puberty (sexually dimorphic); slowly decreasing activity upon completion of longitudinal growth	*Rodents*: slow decrease of neurogenesis with age *Humans*: dramatic postnatal decrease in SVZ cell production and delivery to the olfactory bulb; substantial stabilization in SGZ	
Reparative/regenerative capacity	*Extensive* in fracture healing driven by the periosteal niche	*Limited* to the neurogenic sites and their tissue targets Largely *absent* in most CNS parenchyma
Inter-species differences	*Significant changes across vertebrate phylogeny; mostly conserved niche structure/functions* across most mammals; different chronology of niche activation and cell growth kinetics depending on animals’ lifespan.	*Progressive reductionin spatial distribution and activity* from non-mammalian vertebrates to mammals; *V-SVZ*: early reduction in humans); *SGZ*: relatively constant through species
Stem cell behavior *in vivo* vs. *in vitro*	*Great differences* between *in vitro* and *in vivo* plasticity BMSCs and SSCs are easily isolated *in vitro*, highly expandable, multilineage potential. Extensive (though controversial) trans-differentiation potential; exclusive osteolineage fate *in vivo*	*Great differences* in differentiative fate between *in vitro* (multipotent) and *in vivo* (mainly neuronal) conditions Isolation through neurospheres (V-SVZ) or monolayer (SGZ) of highly expandable primary progenitors

*Dashed areas refer to parameters which strongly (dark gray) or slightly (light gray) differ between the two systems*.

*V-SVZ, ventricular-subventricular zone; SGZ, subgranular zone; BM, bone marrow; e.c.m., extracellular matrix;COL2, type II collagen; ACAN, aggrecan; OP, osteopontin; OC, osteocalcin; ON, osteonectin; MPCs, mesodermal progenitor cells; GFAP, glial fibrillary acidic protein; DCX, doublecortin; PSA-NCAM, polysialylated form of the neural cell adhesion molecule; OPCs, oligodendrocyte precursor cells*.

*Note: the table content is referred only to non-hematopoietic cell components of the bone marrow niche, which are those involved in the formation of most bone precursors and stromal cells, and only indirectly involved in hematopoiesis, by supporting HSC homeostasis and maintenance*.

*^a^For thorough discussion of MSC and NSC secretome, see Salgado et al. (2015) and Drago et al. (2013)*.

In most mammals, bone activity changes during the entire lifespan of an individual, due to modification in the composition of the osteogenic niches. Cellularity decreases with age in all domains of the niche, as a consequence of reduced renewal of both BMSCs and early progenies (Muschler et al., 2001; Ochareon and Herring, 2011; Schlecht et al., 2014), BMSC plasticity being also impaired (Zhou et al., 2008; Choumerianou et al., 2010; Asumda and Chase, 2011).

## Neural Stem Cells and Their Neurogenic Niches

For a long time, the adult mammalian CNS has been considered unable to undergo cell renewal, since it is composed of “perennial” nerve cells (Colucci-D’Amato et al., 2006). Yet, populations of NSCs actually persist in some adult CNS regions (Reynolds and Weiss, 1992), producing undifferentiated neuronal and glial precursors (Gage, 2000; Kriegstein and Alvarez-Buylla, 2009; Table 1). Two brain areas generate new neurons that functionally integrate into neural circuits: the forebrain ventricular-­subventricular zone (V-SVZ, or SVZ), the largest germinal region in the adult mammalian brain gives rise to olfactory bulb interneurons (Silva-Vargas et al., 2013); the subgranular zone (SGZ) of the hippocampus generates granule cells in the dentate gyrus (Aimone et al., 2014).

**Figure 1:**
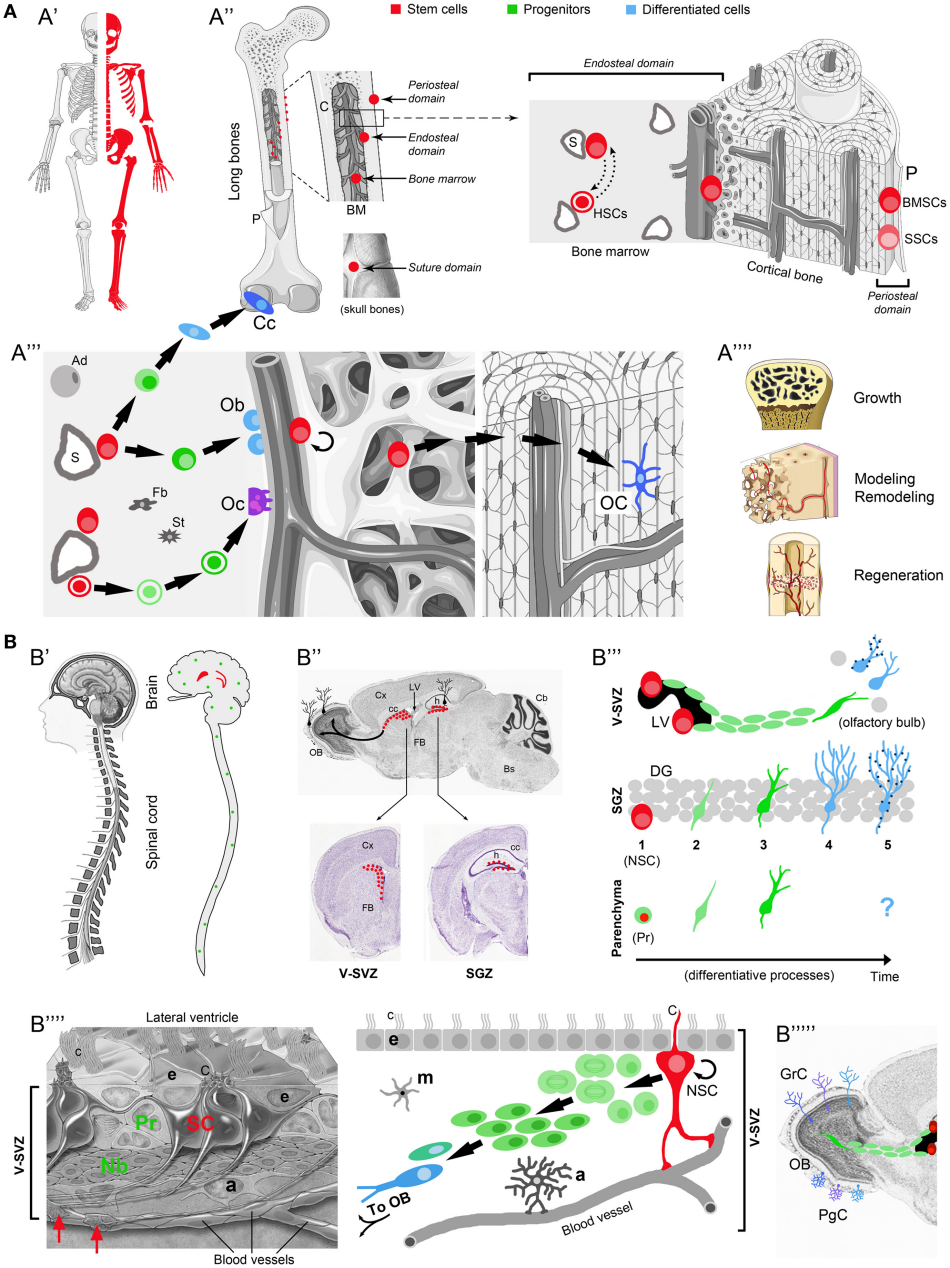
**Comparison between osteogenic and neurogenic niches**. Localization and distribution in the body (A′-B′); localization and distribution in the organ (A′′-B′′); niche components and their reciprocal relationships (A′′′-B′′′′); final outcome in osteogenic/neurogenic (A′′′′-B′′′′′) and growth/regenerative processes (A′′′′). **(A)** Osteogenic niche. A′, All skeletal bones contain osteogenic niches through most of their extension; A′′, in most bones these niches can be found in periosteal, endosteal, and bone marrow (BM) position; in the skull, they occupy the suture domains; P, periosteum; BMSCs, bone marrow stromal cells; SSCs, skeletal stem cells; HSCs, hematopoietic stem cells; S, sinusoids; dotted lines with head arrows indicate reciprocal influence between BMSCs and HSCs. A′′′, Histological organization, cell components, lineage, and cell interactions in the osteogenic niche (endosteal domain); Ob, osteoblasts; Cc, chondrocytes; green cells: intermediate progenitors (osteoblast, chondroblasts, osteoclast, progenitors, macrophages); Oc, osteoclasts; OC, osteocytes; Ad, adipocytes; St, stromal cells; Fb, fibroblasts. A′′′′, Different outcomes from osteogenic stem cells involve both homeostatic cell renewal and lesion-induced regeneration (modified from “Slide kit Servier Medical Art,” www.servier.com). **(B)** Neurogenic niche. B′ Two canonical neurogenic niches do contain stem cells in the brain (here represented in humans, their number and location being similar in mammals), and produce functional neurons for specific regions; parenchymal progenitors also divide throughout the CNS (green dots; not represented in B′′), yet giving rise to “incomplete” neurogenesis and gliogenesis (see B′′′). B′′, SVZ and SGZ niches on the wall of the lateral ventricles and in the dentate gyrus of the hippocampus (represented in mice; for differences in humans see Table 1); top, sagittal section; bottom, coronal sections; images from Allen Brain Atlas (Website: © 2015 Allen Institute for Brain Science. Allen Mouse Brain Atlas [Internet]; available from: http://mouse.brain-map.org.); Cx, cerebral cortex; cc, corpus callosum; OB, olfactory bulb; LV, lateral ventricle; h, hippocampus; Cb, cerebellum; FB, forebrain; Bs, brainstem. B′′′, Cell lineage and displacement; in canonical neurogenic sites (SVZ and SGZ) complete neurogenesis involves: dividing stem cells (SC) (1), secondary progenitor cells or neuroblasts (2), immature neurons (3), mature neurons (4), and fully integrated, functional neurons (5) (dark blue dots indicate the establishment of synaptic contacts). In non-canonical neurogenic sites (CNS parenchyma), only incomplete neurogenesis occurs, starting from parenchymal progenitors (Pr) and giving rise to a progeny of immature cells with apparently no further outcome [modified from Bonfanti and Peretto (2011)]. B′′′′, Left: histological organization of the SVZ neural stem cell niche; right: cell components, lineage, and cell interactions in the neurogenic niche. NSC, neural stem cell; Pr, progenitors (transit-amplifying cells); Nb, neuroblasts (forming chains which exit the SVZ by tangential migration); a, astrocytes; m, microglia; e, ependyma; c, cilia; C, radial glia-like cilium; red arrows, contacts between stem cell processes and blood vessels [modified from Mirzadeh et al. (2008)]. B′′′′′, Specific subpopulations of interneurons, e.g., granule cells (GrC) and periglomerular cells (PgC), functionally integrate in the olfactory bulb. Note the striking differences emerging in the two systems by comparing the extremes in **(A,B)** (A′ vs. B′, A′′′′ vs. B′′′′′; see text).

In the adult SVZ, NSCs are a population of special cells with certain astrocyte properties, which contact the ventricle with an apical process surrounded by ependymal cells forming pinwheel-like structures (Mirzadeh et al., 2008; Figure 1). They give rise to intermediate progenitors (transit-amplifying cells; Doetsch et al., 1999), the majority of which are actively cycling. These progenitors divide on average three times (during 3–4 days) before differentiating into neuroblasts, a half of which then divide at least once in the SVZ (Ponti et al., 2013). In most mammals, neuroblasts reach the olfactory bulb through “tangential chain migration,” by sliding past each other in specific tunnels formed by an astrocytic meshwork (Lois et al., 1996; Peretto et al., 1997). About 10,000 new neurons are generated daily in the mouse SVZ (Ponti et al., 2013), half of which will die before functional integration (Petreanu and Alvarez-Buylla, 2002; Winner et al., 2002), the survivors differentiating into subsets of olfactory bulb interneurons (Obernier et al., 2014). Only small numbers of oligodendrocytes are generated *in vivo* (Menn et al., 2006), whereas in culture, after expansion of the NSC population, most of the progeny acquires aglial (mainly astrocytic) fate, with only 10–20% of neurons (Gritti et al., 2009).

In the SGZ, new neurons arise from two populations of primitive cells (radial – NSCs – and horizontal, slowly dividing cells; Ming and Song, 2011). Similarly to SVZ, they give rise to rapidly amplifying progenitor cells, which divide less than three times (Berg et al., 2015), and then in the next few weeks differentiate into immature neurons developing dendritic arborizations and axonal projections, then beginning to receive excitatory input from cortical perforant path axons (Vadodaria and Gage, 2014; Yu et al., 2014). Unlike SVZ neuroblasts, the hippocampal granule cell precursors perform a very short tangential and then radial migration, confined within the dentate gyrus.

The embryonic origin of the neurogenic niches is strictly linked to the proliferative activity of germinative layers, in periventricular position. The whole CNS forms by radial migration of the progeny from these layers, which mostly disappear postnatally. During development, the neurepithelium is in contact with both the ventricular and pial surfaces of the brain; then, as thickness increases, these cells transform into *radial glia*, a population of astrocytic precursors not only acting as scaffold for migrating neurons but also behaving as multipotent SCs (Malatesta et al., 2000; Noctor et al., 2001). Postnatally, quiescent radial glia-like cells persist as astrocytic-like SCs within remnants of the germinal layers (Tramontin et al., 2003; Merkle et al., 2004; Peretto et al., 2005; Yu et al., 2014; Nicola et al., 2015). In the SVZ, the SC process opposite to that “fishing” in the ventricle contacts the vasculature (Mirzadeh et al., 2008; Figure 1). Also, transit-amplifying cells directly contact blood vessels at specialized sites that lack glial and pericyte coverage (Shen et al., 2008; Tavazoie et al., 2008). Basal lamina structures extending from blood vessels to the ependymal layer do contact cells at each stage of the lineage, binding growth factors (Mercier et al., 2002). In the SGZ, angiogenesis accompanies neurogenesis (Palmer et al., 2000), whereas the vascular bed is largely quiescent in SVZ. SC activity in the neurogenic niches is finely regulated by various signals involving growth factors, morphogens, cell–cell interactions, neurotransmitters, and endothelial signals (Tong and Alvarez-Buylla, 2014; Table 1). The whole process, from SC proliferation to neuronal integration, can be modulated by internal (hormones, trophic factors) and external (environmental) stimuli.

Both mammalian neurogenic niches show differences related to species and ages (Bonfanti and Peretto, 2011). The rostral migratory stream is active throughout life in rodents but temporally restricted to the first 18 months in humans (Sanai et al., 2011; Wang et al., 2011). By contrast, postnatal neurogenesis occurring in transient germinal layers of the cerebellum does persist in adult rabbits (Ponti et al., 2008). Unlike mammals, in which adult neurogenesis occurs mostly within two “canonical” neurogenic zones, in non-mammalian vertebrates NSCs and neurogenesis are widespread through many CNS regions (Zupanc, 2006; Grandel and Brand, 2013). During the last few years, new examples of cell genesis, involving neurogenesis and gliogenesis, have been shown to occur in adult parenchymal regions of the mammalian CNS (Bonfanti, 2013; Feliciano et al., 2015), where dividing progenitors have been detected, suggesting that *de novo* neural cell genesis could be more widespread than previously thought (Nishiyama et al., 2009; Migaud et al., 2010; Bonfanti and Peretto, 2011). Yet, in most cases of parenchymal neurogenesis, the newly generated cells live only transiently and do not integrate in neural circuits, their role remaining obscure (Bonfanti and Peretto, 2011; Feliciano et al., 2015). Taken together, the highly restricted localization of adult neurogenesis in mammals underlines its exceptional character with respect to the genetically determined connectivity typical of most CNS tissue, which remains substantially refractory to cell renewal and regeneration.

## Similarities and Differences Between Osteogenic and Neurogenic Systems

By comparing osteogenic and neurogenic SC niches a few similarities and significant differences emerge, concerning the relationships between SCs and the tissue/organ they belong to (Table 1; Figure 1). In both niches, close connections with blood vessels are observed, since blood-derived nourishment and signaling is vital to niche homeostasis. NSCs and BMSCs also share non-specific markers, such as the cytoskeletal protein nestin, a basic structural element in mitotically active cells, along with molecular signals which exert pleiotropic functions in development and homeostasis (e.g., Wnt, BMP, and Notch).

The most evident differences between osteogenic and neurogenic niches/systems are represented in the extremes of Figures 1A,B: abundant availability of widely distributed SCs/niches in bones (A′) grant continuous renewal and lesion-induced regeneration throughout the skeleton (A″″), whereas highly restricted SCs/niches in the CNS (B′) only allow the renewal of well specified neuronal populations (B″″′) (Obernier et al., 2014). In the whole mammalian body, the number and distribution of SC niches are highly heterogeneous, spanning from millions of “multiple, disperse” niches in blood, skin, and intestine (Nystul and Spradling, 2006), to only two niches capable of “complete” neurogenesis in the adult brain (Bonfanti and Peretto, 2011). These differences drive important consequences since multiple niches will allow homeostatic cell renewal and injury-induced regeneration in many tissues, whereas most brain regions are substantially non-renewing/non-regenerating (Bonfanti, 2011). Based on niche number, dislocation and rate of cell renewal, bone may be considered a borderland, given that osteogenic SCs are found throughout the skeleton. Accordingly, upon fracture, resident stromal, stem/progenitor cells, working in tandem with macrophages and circulating blood cells, lead to scarless healing (Colnot, 2009; Park et al., 2012). The mammalian CNS, in spite of its NSC content and intrinsic plasticity of neuronal and glial elements, shows very low and restricted rate of cell renewal, being hardly capable of repair from extensive damage or neuronal loss (Weil et al., 2008). NSC niches are deeply isolated within the most complex organ of the body, providing homeostatic replacement/addition of neurons only within restricted areas. Outside the neurogenic regions, in addition to the lack of SC niches, the substantial failure in CNS repair is due to evolutionary constraints, including incapability to recapitulate developmental pathways and strong immune reaction leading to necrosis instead of regeneration (Weil et al., 2008; Bonfanti, 2011). For these reasons, in spite of significant progress obtained in biomedical research, rational translation of the enormous body of basic research to the clinics is still very limited.

## Cell–Tissue Specificity and Translational Issues

It seems clear that SCs in the two niches originate from distinct embryo layers (except from skull SSCs), then adapt to utterly different morpho-functional environments: NSCs occupy topographically precise positions within specific neural systems, whereas BMSCs/SSCs, similarly to HSCs, balance free movement and stable positions. Hence, the general idea of using BMSCs as a regenerative treatment applied to CNS disorders is far from being substantiated. On the other hand, many studies support the evidence that BMSCs (as well as other MSC types) can produce beneficial – bystander – effects through the secretion of immune modulatory or neurotrophic paracrine factors (Martino et al., 2011; Drago et al., 2013). Nevertheless, the exact mechanisms underlying such effects are still far from being fully elucidated. Phase I–II clinical trials for neurological disorders (multiple sclerosis, amyothrophic lateral sclerosis, and spinal cord injury) suggested that autologous BMSCs inoculation is safe and feasible and may induce systemic immunomodulatory effects explaining moderate clinical improvements. Conversely, no clear sign of neurodegeneration induced by cell replacement mechanisms could be investigated in any case, to date (Squillaro et al., 2015).

In addition, the heterogeneity of the BM stroma, in terms of cellular composition, is often neglected in the design of experimental cellular treatments, especially when minimal tissue manipulation (i.e., harvesting/fractionation and direct implantation, without prior culture amplification) is performed. It is worth noting that BMSC implantation experiments clearly indicated that the range of tissues which can be actually generated *in vivo* from both SSCs and BMSCs exclusively involves those making up the skeleton (Sacchetti et al., 2007; Bianco et al., 2013a,b; Tasso et al., 2013). Hence, the realistic translational consequences of *in vitro* BMSC plasticity are more limited than supposed, while their plausible trophic effect, in selected tissue environments, may be due to the innate role of BMSCs (and other MSCs) in forming supporting stroma in mesodermal-derived tissues, and to their rich secretome, exerting autocrine and paracrine effects (Kim et al., 2013; Tran and Damaser, 2015). On these bases, future studies should be aimed first at obtaining full understanding of the “natural” SC niche dynamics (paying attention to differences between tissues and species) and, second, at further elucidating the nature of cell-to-cell and molecular interactions adopted by different types of SCs in each physiological/pathological environment, allowing possible therapeutic outcomes.

## Conflict of Interest Statement

The authors declare that the research was conducted in the absence of any commercial or financial relationships that could be construed as a potential conflict of interest.
